# Mass Spectrometry-Inspired Degradation of Disinfection By-Product, 2,6-Dichloro-1,4-benzoquinone, in Drinking Water by Heating

**DOI:** 10.5702/massspectrometry.A0068

**Published:** 2018-06-29

**Authors:** Jiying Pei, Ruiling Zhang, Chengchih Hsu, Yinghui Wang

**Affiliations:** 1 School of Marine Sciences, Guangxi University, Nanning 53004, P. R. China; 2 Department of Chemistry, National Taiwan University, Taipei 10617, Taiwan

**Keywords:** heat degradation, disinfection by-product, 2,6-dichloro-1,4-benzoquinone

## Abstract

2,6-Dichloro-1,4-benzoquinone (DCBQ), a highly toxic and carcinogenic disinfection by-product, was degraded during the electrospray process by elevating the source temperature. This unexpected finding inspired us to use heating to degrade DCBQs in drinking water. The results show that about 99% of DCBQs in the drinking water were degraded in one minute by heating to 100°C with room light irradiation. Therefore, a conclusion can be drawn that heating enables the degradation of DCBQs in drinking water.

## INTRODUCTION

Halobenzoquinones (HBQs), which emerge as a new class of disinfection by-products (DBPs) in drinking water,^1,2)^ are the consequence of common disinfection methods, such as chlorination or chloramination. HBQs, especially 2,6-dichloro-1,4-benzoquinone (DCBQ) commonly detected in drinking water,^3)^ are predicted to be toxicologically relevant on the basis of their lowest observed adverse effect levels (LOAELs) with quantitative structure toxicity relationship (QSTR) analysis.^4)^ The LOAEL of DCBQ is 0.049 mg/kg·day, which is 1000 times lower than most of the regulated DBPs.

Ultraviolet (UV) irradiation^5,6)^ and chemical oxidation technology^7,8)^ are two effective pathways for water disinfection treatment. It is reported that over 80% of initial HBQs could be removed at typical UV doses used in water treatment plants (WTPs).^9)^ Though UV disinfection is effective in removal of organic pollutant in drinking water, an online survey shows that 44% of USA WTPs have not introduced UV disinfection into the water treatment processes.^10)^ To decrease daily exposure to DCBQs, more easy-to-operate methods are still needed to degrade DCBQs in various water sources.

During DCBQ analysis with mass spectrometry (MS), we accidently found that DCBQs were degraded during the electrospray process when the ionization source temperature was elevated. Inspired by this, we tried to use heating to degrade DCBQs in water, and it proved that the method was feasible.

## EXPERIMENTAL

### Reagents and solutions

Methanol was purchased from Honeywell Burdick & Jackson Inc. (USA). Formic acid (FA), ammonium acetate and DCBQ were obtained from Sigma-Aldrich Chemical Co., Ltd. (USA). All the reagents were used directly without any further purification. Distilled water (18.2 MΩ) was produced by Milli-Q system (Millipore Inc., Bedford, MA, USA). DCBQ standard stock solution (1000 μg mL^−1^) was prepared by dissolving DCBQ into methanol, and stored at −20°C. The DCBQ solutions in the heat and light degradation experiment were prepared by diluting DCBQ stock solution with water, and the methanol content was 0.4% with DCBQ concentration of 4 μg mL^−1^.

### Heat-induced DCBQ degradation in the drinking water

Two tap water was collected from laboratory using 1 L amber glass bottles precleaned with methanol and water, and twenty-five nanograms of DCBQs were spiked into the two group of 500 mL of tap water. One water sample was subjected to heat to boiling while the other one was placed at room temperature. 0.25% FA was added to keep the samples in an acidic condition before solid phase extraction (SPE), which was to avoid DCBQ degradation in neutral or alkaline conditions.

### Solid phase extraction of samples

The extraction procedures referred to Zhao’s method.^2)^ Waters Oasis HLB cartridges (6 mL, 200 mg per cartridge; Milford, MA) used for SPE were first conditioned with 6 mL methanol containing 0.25% FA and subsequent 6 mL acidified water with 0.25% FA. Then 500 mL water sample was loaded with a flow rate of 4 mL min^−1^. After sample loading, the cartridges were washed with 6 mL acidified water (with 0.25% FA) and 6 mL methanol/water (v/v 50/50 with 0.25% FA), and then were dried for 10 min under vacuum. The analytes on the cartridge were eluted with 6 mL methanol (with 0.25% FA). The volume of each eluent was reduced to 0.25 mL under a gentle nitrogen stream at 35°C. Finally the concentrated samples were reconstituted with water (with 0.25% FA) to a final volume of 0.5 mL (water/methanol, v/v 50/50 with 0.25% FA). The recovery was calculated by the standard addition method. With twenty-five nanograms of DCBQs being added into 500 mL of tap water, the recoveries were determined by: 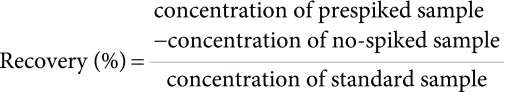 which was detected to be 61%.

### Instrumentation

#### Liquid chromatography

An Agilent 1290 series LC system consisting of a binary pump and an autosampler was used for LC separation with an Agilent Zorbax Eclipse Plus C18 column (50×2.1 mm i.d., 1.8 μm; USA) at 35°C. The flow rate of the mobile phase was 0.2 mL min^−1^, and the injection volume was 2 μL. The initial mobile phase was 30% A (methanol) and 70% B (water containing 5 mmol L^−1^ ammonium acetate and 0.25% FA), followed by a linear gradient to 90% A in 5 min, and kept isocratic in 2 min, and then back to 30% A in 0.5 min.

#### Mass spectrometry

A triple quadrupole tandem mass spectrometer (Agilent 6460, USA) equipped with an electrospray ionization (ESI) source was used throughout the experiment. The MS detection was operated in negative ionization mode combined with full scan and multiple reaction monitoring (MRM). The ionization source parameters were set as follows: spray voltage, −3500 V; curtain gas flow, 8 L min^−1^; nebulizer gas flow, 40 psi. The curtain gas temperature was set as 150°C unless otherwise specified. Compound-dependent parameters including fragment energy (FE) and collision energy (CE) are presented in Table S1 in the supplementary material.

The calibration curve for the quantitation of DCBQ with LC-MS/MS was shown in Fig. S1 with the limit of detection being 4.1 μg L^−1^.

## RESULTS AND DISCUSSION

### Discovery of DCBQ degradation during electrospray ionization mass spectrometry

**Figure 1:**
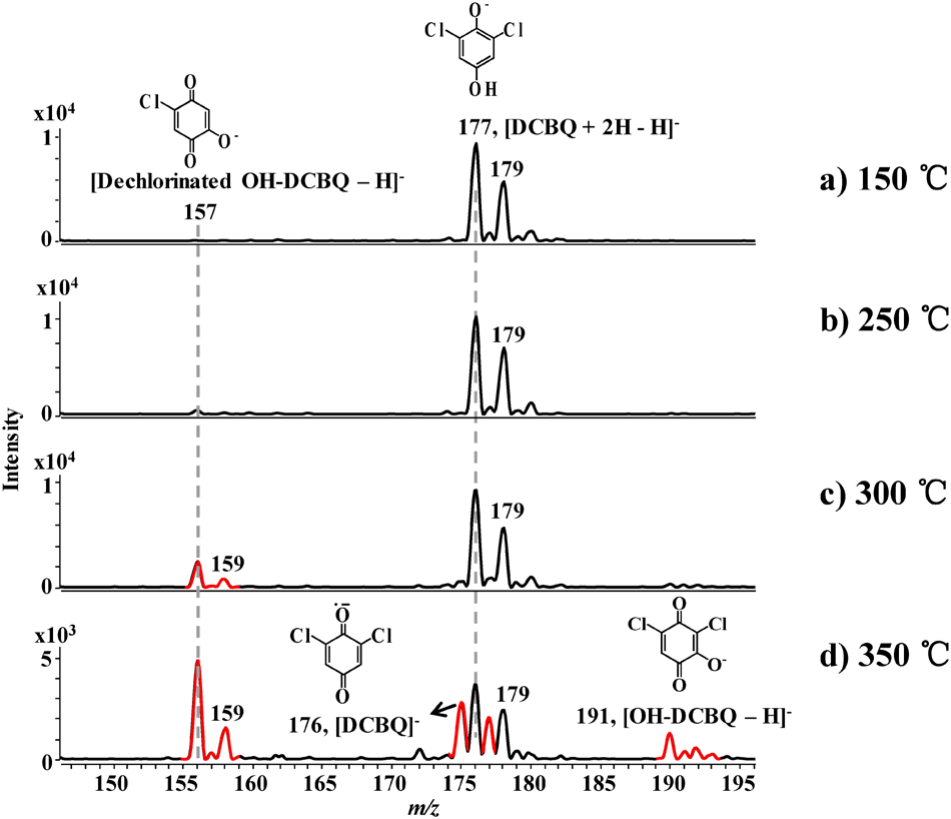
Fig. 1. Degradation of DCBQs (4 μg mL^−1^) ([M+H]^−^, *m*/*z* 177; M^−^, *m*/*z* 176) under different ESI source temperatures. a) 150°C, b) 250°C, c) 300°C, d) 350°C.

Li’s group found the production of [M+H]^−^ ions of DCBQs in negative ESI-MS,^1)^ and elevation of the ESI source temperature favored the formation of M^−^ ions and the suppression of [M+H]^−^ ions. The authors speculated that the unexpected reduction of DCBQs in negative ESI mode might be related with the electrochemical reaction at the spraying tip.^11,12)^ However, our previous work showed that corona discharge was also responsible for analyte redox in ESI.^13,14)^ Moreover, we have illustrated that the unexpected reduction of quinones and their derivatives in both positive and negative modes was related to corona discharge.^15,16)^ To further explore the mechanism of DCBQ reduction in negative ESI, we conducted the same temperature elevation-experiment in an Agilent LC-MS/MS instrument. It was unexpectedly found that the signal intensities of the species with *m*/*z* 157 and 191 were increased when the ESI source temperature was elevated from 150°C to 350°C, accompanying the increase of the signal intensity of M^−^ ions and the decrease of the signal intensity of [M+H]^−^ ions (Fig. 1). According to the MS/MS spectra (see Fig. S2†), the species with *m*/*z* 157 and 191 were identified to be dechlorinated 3-hydroxyl-2,6-dichloro-1,4-benzoquinone (dechlorinated OH-DCBQ) and OH-DCBQ, respectively. Qian *et al.* reported that ultraviolet radiation could lead to the degradation of DCBQs in water with the degradation products being OH-DCBQ, dechlorinated OH-DCBQ and halo-benzenetriols (*m*/*z* 193).^9)^ Since the degradation products of DCBQs during ESI are similar with those observed with ultraviolet radiation, it inspired us to use heating to degrade DCBQs in water.

### Heat- and light-induced degradation of DCBQs

**Figure 2:**
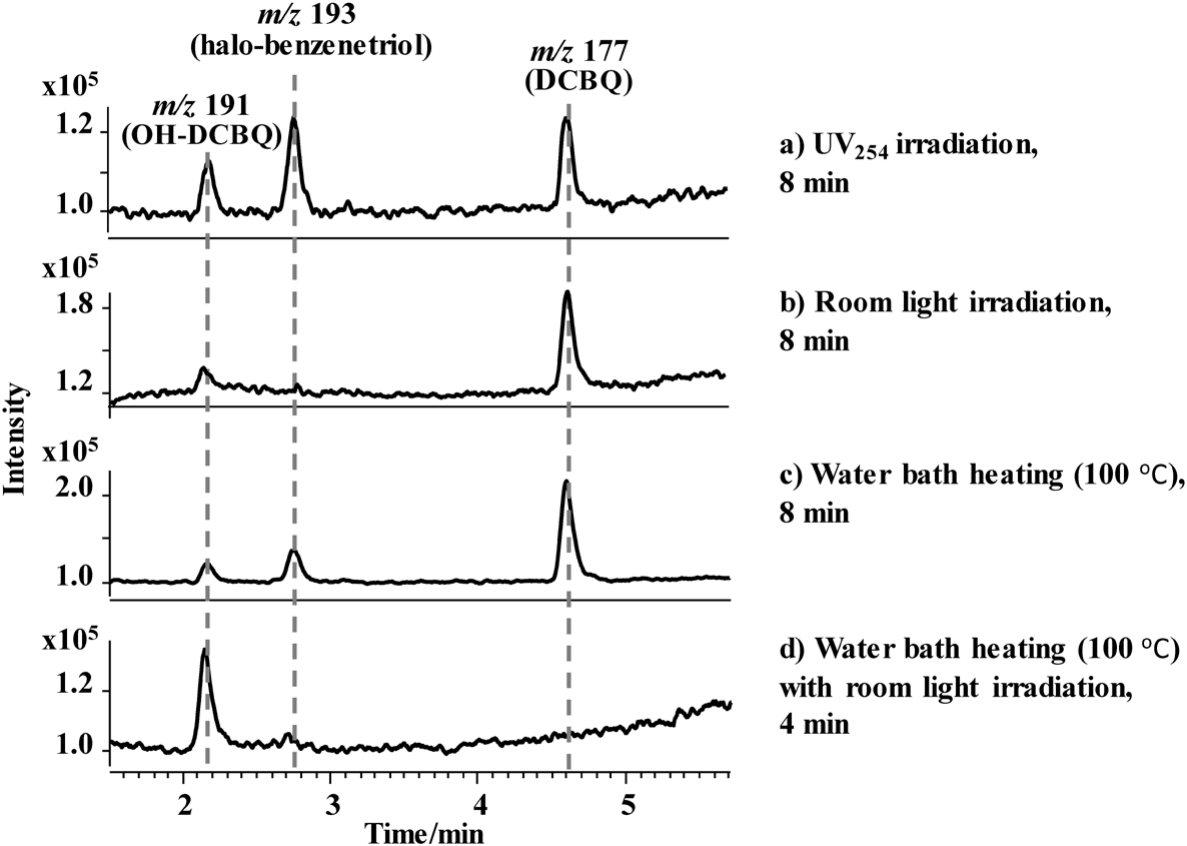
Fig. 2. Chromatograms of DCBQ solutions (4 μg mL^−1^ in water) analyzed by LC-MS/MS after exposure to different conditions. a) UV_254_ irradiation, b) room light irradiation, c) water bath heating (100°C), and d) water bath heating (100°C) with room light irradiation. The solutions for UV_254_ and room light irradiation experiment were exposed to room temperature condition, and the solution for water bath heating only was placed in a brown vial to avoid light irradiation.

**Figure 3:**
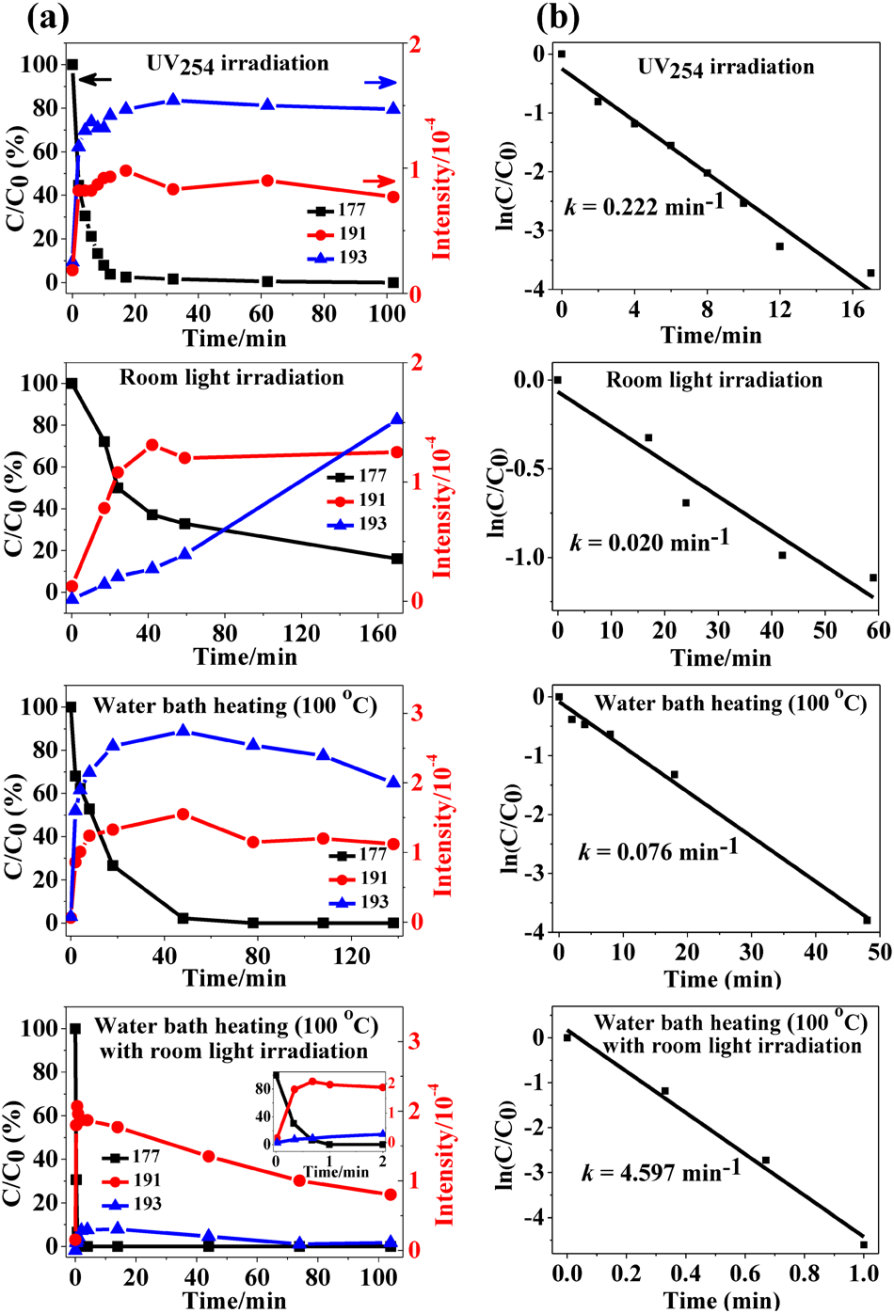
Fig. 3. (a) Dynamics curves and (b) kinetic linear simulation of DCBQ (4 μg mL^−1^ in water) degradation under different conditions (UV irradiation, room light irradiation, water bath heating (100°C), and water bath heating (100°C) with room light irradiation).

Since DCBQ degradation is also related with light irradiation, we simultaneously investigated the effects of heating and light irradiation on DCBQ degradation. Four DCBQ aqueous solutions with the concentrations of 4 μg mL^−1^ were subjected to different conditions. One solution was exposed to water bath heating (100°C) with room light irradiation for 4 min, whereas the other three solutions were subjected to UV_254_ irradiation (irradiated with a 16-W portable UV lamp at 0.004 m^3^ confined dark space), room light irradiation, and water bath heating (100°C) for 8 min respectively. The solutions for UV_254_ and room light irradiation experiment were exposed to room temperature condition. It is worth noting that the solution subjected to water bath heating (100°C) was placed in a brown vial to avoid light irradiation. After treatment of the four solutions under different conditions, the concentrations of DCBQs and the degradation products were analyzed by LC-MS/MS. Because high ESI source temperature led to the degradation of DCBQs during the analysis process, the source temperature was set as 150°C. OH-DCBQ and halo-benzenetriol (*m*/*z* 193) were produced accompanying the degradation of DCBQs under various conditions (Figs. 2, 3a). The degraded products were different from those (OH-DCBQ and dechlorinated OH-DCBQ) observed in the gas phase during elevation of ESI source temperature. This might be related with the different decomposition mechanism of DCBQ in the gas phase (accompanying with corona discharge with the application of high spray voltage) and in the liquid phase.^15,16)^ About 98% of DCBQs were degraded in 17 min after UV_254_ irradiation, whereas only 28% and 73% were degraded in 17 min after room light irradiation and heating treatment respectively. However, over 99% of DCBQs were degraded in 1 min after heating and room light irradiation (Fig. 3a). The kinetic linear simulation indicated that DCBQ degradation followed the first-order kinetics and the degradation rates under various conditions were as follows: water bath heating with room light irradiation (rate constant of DCBQ degradation, *k*=4.597 min^−1^)>UV_254_ irradiation (*k*=0.222 min^−1^)>water bath heating (*k*=0.076 min^−1^)>room light irradiation (*k*=0.020 min^−1^) (Fig. 3b). Besides, we proved that microwave heating also enabled the degradation of DCBQs in water (Fig. S3). As a whole, both heating and light irradiation enable DCBQ degradation.

Qian *et al.*^9)^ proposed that the formation of hydroxylated HBQs during photoreaction was possibly due to the reaction of excited HBQ–water complex with ground state HBQs.^17)^ With continuous UV irradiation, OH-HBQs could further transform to dehalogened OH-HBQ by losing a chlorine or bromine through a substitution reaction. On the other hand, Zhu *et al.* reported the substitution reaction of tetrachloro-1,4-benzoquinone by the replacement of chlorine atom with OH^−^ at room temperature,^18)^ and the reaction rate would be accelerated by the elevated reaction temperature according to Arrhenius equation. Therefore, the degradation rate of DCBQ would be much more improved by the simultaneous application of light irradiation and high temperature, possibly with both the degradation mechanisms.

### Degradation of DCBQs in the drinking water by heating

**Figure 4:**
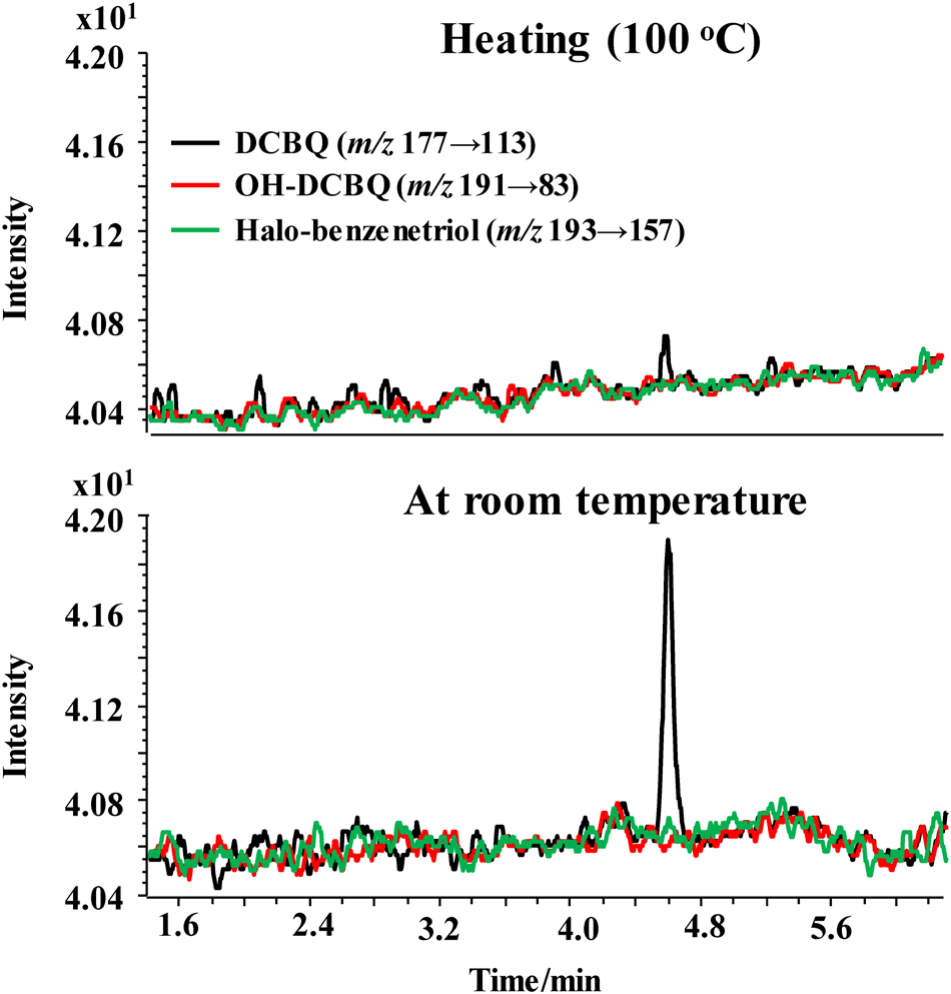
Fig. 4. Degradation of DCBQs in the drinking water a) with heating (100°C) and b) at room temperature. The authentic concentrations of DCBQs in the drinking water for the two solutions after SPE were <0.03 μg mL^−1^.

Then we tested the degradation efficiency of DCBQs in the drinking water by heating. Twenty-five nanograms of DCBQs were spiked into two group of 500 mL of tap water. One water sample was exposed to heating with room light irradiation for 5 min, and the other one was placed at room temperature as control. After the water samples were concentrated by SPE, the concentrations of DCBQs and the degradation products were analyzed by LC-MS/MS. The results show that all of DCBQs, OH-DCBQs and halo-benzenetriols were barely detected in the tap water after heating treatment whereas DCBQs in the tap water at room temperature still existed (Fig. 4). Therefore, it suggests that heating can efficiently remove DCBQs from drinking water.

## CONCLUSION

In conclusion, heating was illustrated to enable the degradation of DCBQ in water, inspired by the degradation of DCBQs during ESI. For drinking water suffering from heating treatment (100°C), almost all the DCBQs were degraded. Therefore, heating before drinking the tap water is suggested to reduce daily expose to DCBQs.

## Notes

Mass Spectrom (Tokyo) 2018; 7(1): A0068
