# Supplementary material for: Mass Spectrometry-Inspired Degradation of Disinfection By-Product, 2,6-Dichloro-1,4-benzoquinone, in Drinking Water by Heating
Source: Mass Spectrom (Tokyo). 2018 Jun 29;7(1):A0068. doi: 10.5702/massspectrometry.A0068 (PMC6018241; doi:10.5702/massspectrometry.A0068)
Supplement: Table S1 and Figs. S1-S3 [file massspectrometry-7-1-A0068-s001.pdf]

Mass spectrometry-inspired degradation of disinfection by-product, 2,6-dichloro-1,4-benzoquinone, in drinking water by heating

Jiying Pei,[a] Ruiling Zhang,[a] Chengchih Hsu,[b] Yinghui Wang[*,a]

[a]School of Marine Sciences, Guangxi University, Nanning, 53004, P. R. China. E-mail: wyh@gxu.edu.cn

[c]Department of Chemistry, National Taiwan University, Taipei 10617, Taiwan.

Key word:

Heat degradation; disinfection by-product; 2,6-dichloro-1,4-benzoquinone

Table S1 MRM conditions used for LC-MS/MS analysis of DCBQ and the degradation products

Analytes	Transition ion pairs	FE/V	CE/V
DCBQ	177→113[a]	100	15
	177→141	100	10
OH-DCBQ	191→83[a]	110	15
	191→35	110	30
halo-benzenetriol	193→157[a]	110	10
	193→35	110	30

[a]The ion pairs used for quantification

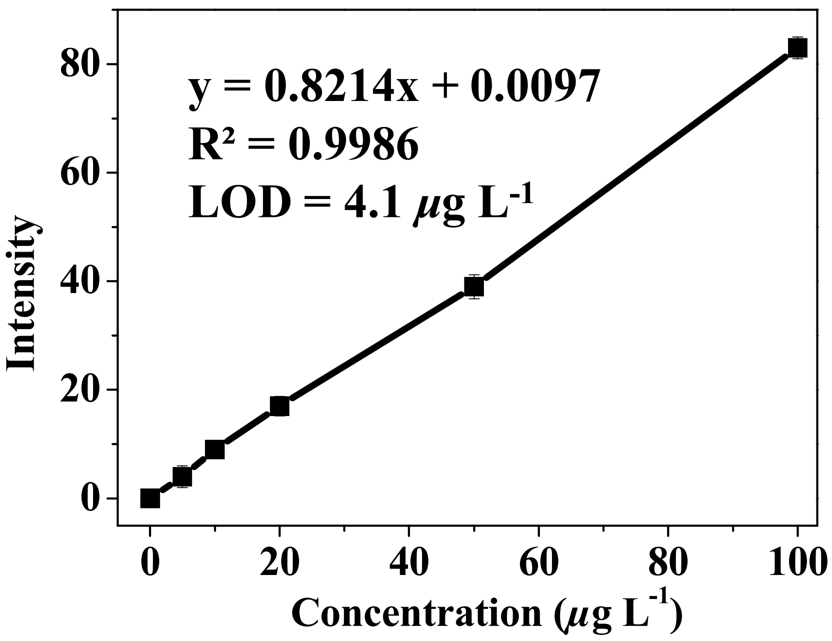

Fig. S1. Calibration curve for DCBQ quantitation by LC-MS/MS

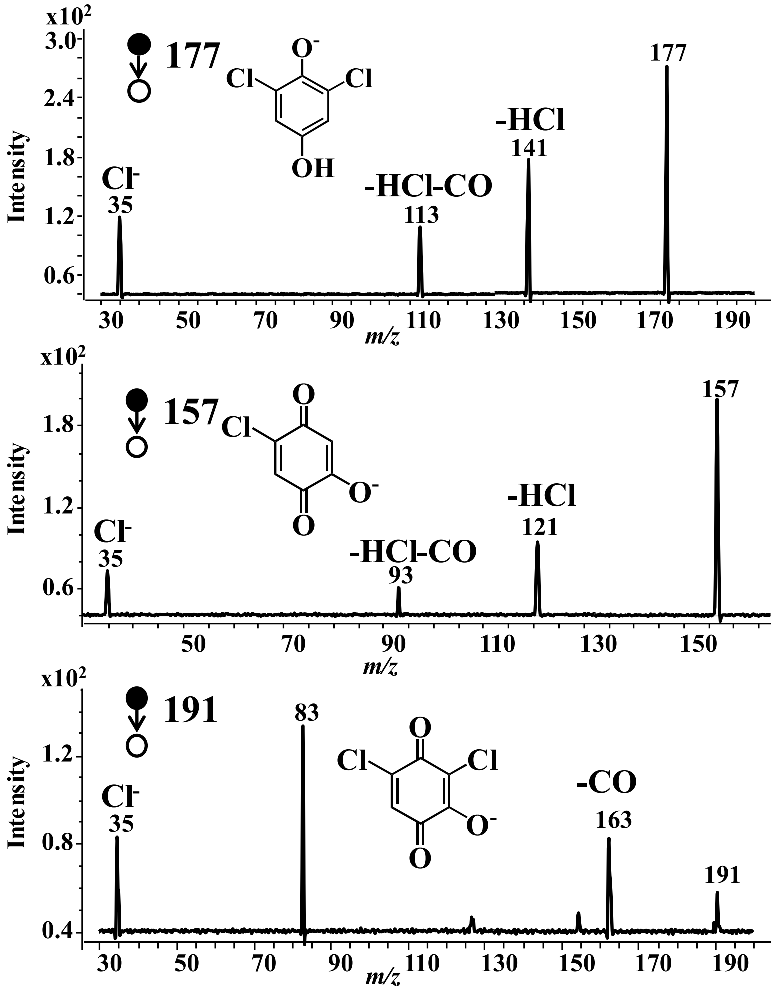

Fig. S2. MS/MS spectra of DCBQ (*m/z* 177), halo-benzenetriol (*m/z* 157) and OH-DCBQ (*m/z* 191).

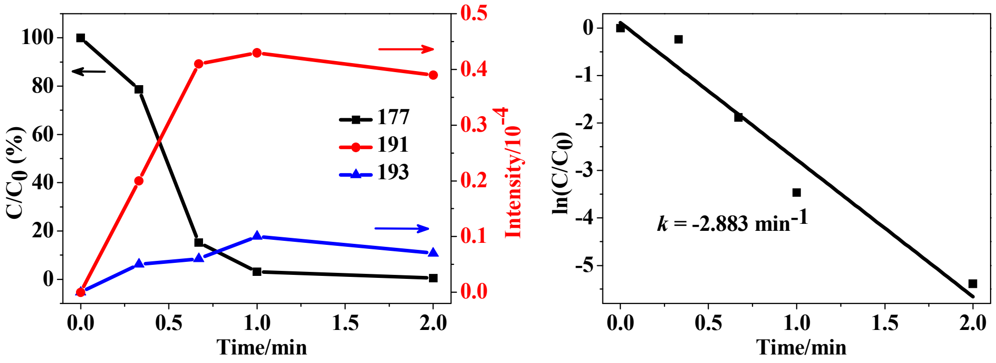

Fig. S3. Degradation dynamics of DCBQs with microwave irradiation.
